# Meta-analysis indicating that high ALCAM expression predicts poor prognosis in colorectal cancer

**DOI:** 10.18632/oncotarget.17707

**Published:** 2017-05-08

**Authors:** Yeqing Zhang, Chunmei Qian, Lin Jing, Jianlin Ren, Yu Guan

**Affiliations:** ^1^ Department of Chinese Internal Medicine, Shanghai Municipal Hospital of Traditional Chinese Medicine, Shanghai University of Traditional Chinese Medicine, Shanghai 200071, China; ^2^ Department of Central Laboratory Medicine, Shanghai Municipal Hospital of Traditional Chinese Medicine, Shanghai University of Traditional Chinese Medicine, Shanghai 200071, China; ^3^ Department of Oncology, Shanghai Municipal Hospital of Traditional Chinese Medicine, Shanghai University of Traditional Chinese Medicine, Shanghai 200071, China

**Keywords:** ALCAM, outcome, clinical features, CRC

## Abstract

Activated leukocyte cell adhesion molecule (ALCAM) has been linked to the development and progress of colorectal cancer (CRC). In this meta-analysis, we examined whether ALCAM expression is predictive of survival outcomes in CRC patients. We included 7 studies with 2048 patients in our meta-analysis after searching the PubMed, Cochrane Library, EMBASE, OVID and Web of Science databases. High ALCAM expression was associated with poor overall survival among CRC patients (HR = 1.94, 95%CI = 1.05–3.58, *P* = 0.03). High ALCAM expression was also associated with aggressive clinicopathological features such as tumor stage (T3,T4/T1,T2; HR = 2.66, 95%CI = 2.01–3.51, *P* < 0.00001), nodal status (Positive/Negative, HR = 2.12, 95%CI = 1.61–2.82, *P* < 0.00001), distant metastasis (M1/M0, HR = 3.30, 95%CI = 2,21–4.91, *P* < 0.00001), tumor grade (grade3/grade1,2, HR = 1,28, 95% CI = 1.00–1.62, *P* = 0.05), and patient age (> 60/< 60, HR = 1.29, 95%CI = 1.01–1.66, *P* = 0.05). These findings indicate that high ALCAM expression is associated with poor prognosis and advanced clinicopathological characteristics in CRC patients.

## INTRODUCTION

Colorectal cancer (CRC) is one of most frequently malignant tumors globally, and only 10–20% of CRC patients are diagnosed early stage [1]. The incidence of CRC has been increasing rapidly and the treatment is extremely limited for advanced CRC [2]. The conventional prognostic factors for CRC are histological tumor grades and disease stages including cell differentiation, depth of tumor invasion, and lymph node metastasis [3, 4]. In recent studies, cell surface markers were identified as potential therapeutic targets [5–7]. Yet, the prognostic value of these putative molecular biomarkers in CRC was ambiguous. Therefore, there is an urgent need to develop molecular diagnostic markers for CRC that would enable effective and early clinical screening and prevention strategies.

Activated leukocyte cell adhesion molecule (ALCAM or CD166) that belongs to the cell surface immunoglobulin superfamily was first described as a CD6 ligand. The ALCAM gene is mapped to human chromosome 3q13 [8, 9]. CD166 is usually expressed in proliferative or trafficking cells like activated leukocytes, embryonic hematopoietic and endothelial cells, lung endothelial cells, endometrial cells, and blastocysts [10]. Modulation of CD166 function inhibits matrix metalloproteinase-2 activation [11], which decreases tumor angiogenesis and the expression of extracellular matrix proteins, thereby altering tumor progression [12]. Also, ALCAM expression has been reported in prostate [13], breast [14], ovarian [15], pancreatic [16], and colorectal [17] cancers. Although many studies have reported the prognostic value of ALCAM expression in CRC [18, 19], the results have been contradictory. This may have been caused by limited study samples in the studies in addition to other factors. Therefore, we conducted a pooled analysis to investigate the clinical significance of ALCAM in CRC and its potential value as a biomarker.

## RESULTS

### Study selection

**Figure 1:**
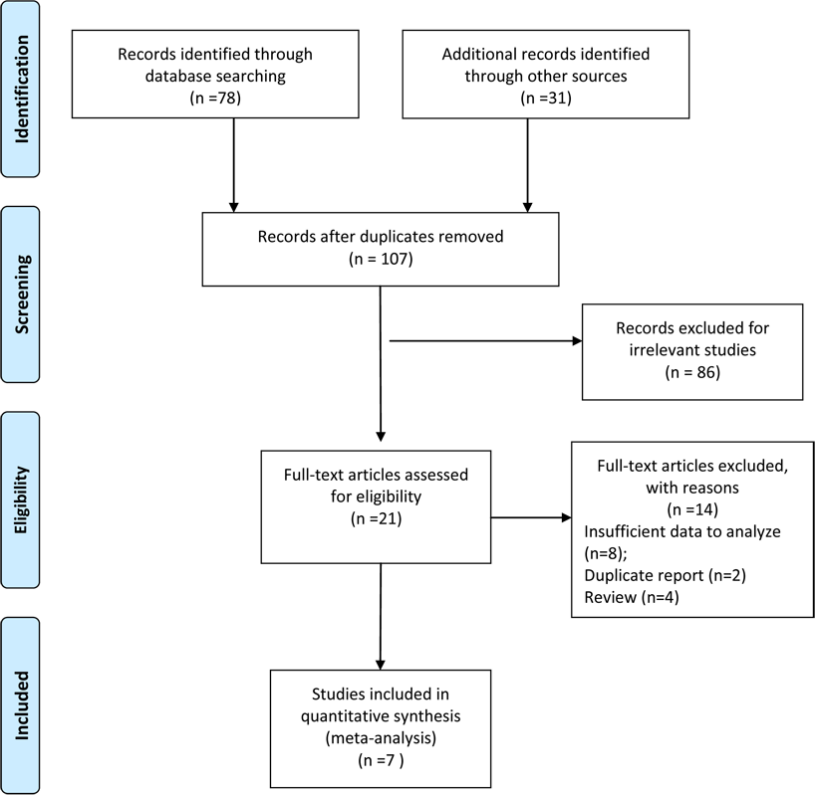
Flow diagram of the study selection process

We retrieved 107 studies after comprehensively searching PubMed, Cochrane Library, EMBASE, OVID, Web of Science databases and other sources. Two duplicate reports were excluded. After detailed screening by title and abstracts, 86 more records were excluded. A full-text assessment of the remaining 21 articles resulted in exclusion of 14 more studies and finally 7 studies that fulfilled the inclusion criteria were enrolled in our meta-analysis [19–25]. A flow chart of the selection process is shown in Figure 1.

### Study characteristics

Table 1 shows details of the 7 studies included in the meta-analysis. A total of 2048 CRC patients were included in this meta-analysis. Among the 7 studies, 3 were from Germany and 2 each from Korea, Australia and Switzerland. The ALCAM expression was measured by tissue microassay (TMA) or immunohihstochemistry (IHC) in the 7 studies. The studies also included overall survival (OS) and disease-free survival (DFS) statistics.

**Table 1:** Baseline characteristics of the studies included

No.	First Author	Year	Country	Sample size	Mean Age (Year)	Duration of Follow-up(month)	Survival Conditions	Testing Methods	Staining Pattern	RR(95%CI)
1	Jerry Zhou	2015	Australia	45	64	NM	OS	TMA	Membrane and cytoplasm staining	5.26 (1.57–17.62)
2	Michael Tachezy	2012	Germany	300	54 (15–91)	39 (1–180)	OS	TMA	Membrane staining	0.61 (0.40–0.93)
3	Sung Hoon Sim	2014	Korea	112	62 (33–82)	48.1	DFS	IHC	Membrane and cytoplasm staining	5.61 (1.82–17.36)
4	A Lugli	2010	Switzerland	1274	69.9 (30–96)	56.4	DFS	TMA	Membrane staining	0.85 (0.56–1.29)
5	Hee Jin Lee	2013	Korea	96	67	NM	OS	IHC	Membrane and cytoplasm staining	3.06 (1.65–5.69)
6	W Weichert	2004	Germany	111	65 (41–87)	47	OS	IHC	Membrane and cytoplasm staining	2.34 (1.10–4.98)
7	David Horst	2009	Germany	110	66.5 (41–92)	94.8 (4.8–162)	OS	TMA	Membrane staining	2.06 (1.22–3.48)

Abbreviations: NM: not mentioned; TMA: tissue microassay; IHC: immunohistochemistry; OS: overall survival; DFS: disease-free survival.

### Quality assessment of studies

Table 2 shows the quality assessment of the 7 included studies by the Newcastle-Ottawa Scale (NOS) grading system taking into account selection, comparability and outcome of each study. The NOS scores for the 7 studies ranged from 6 to 9 and therefore regarded as high quality.

**Table 2:** Quality assessment using the newcastle-ottawa quality assessment scale in the studies

Study (Author, years)	Selection				Comparability	Outcome			Scores
	1	2	3	4		1	2	3	
Jerry Zhou (2015)	★	★	★	−	★★	★	★	-	7
Michael Tachezy (2012)	★	★	−	★	★★	★	★	★	8
Sung Hoon Sim 2014)	★	★	−	★	★	★	−	★	7
A Lugli (2010)	★	★	★	★	★★	★	−	−	7
Hee Jin Lee (2013)	−	★	★	−	★★	−	★	★	6
W Weichert (2004)	★	★	★	★	★★	★	★	★	9
David Horst (2009)	★	−	★	★	★★	★	★	★	8

### Association between ALCAM expression and OS

**Figure 2:**
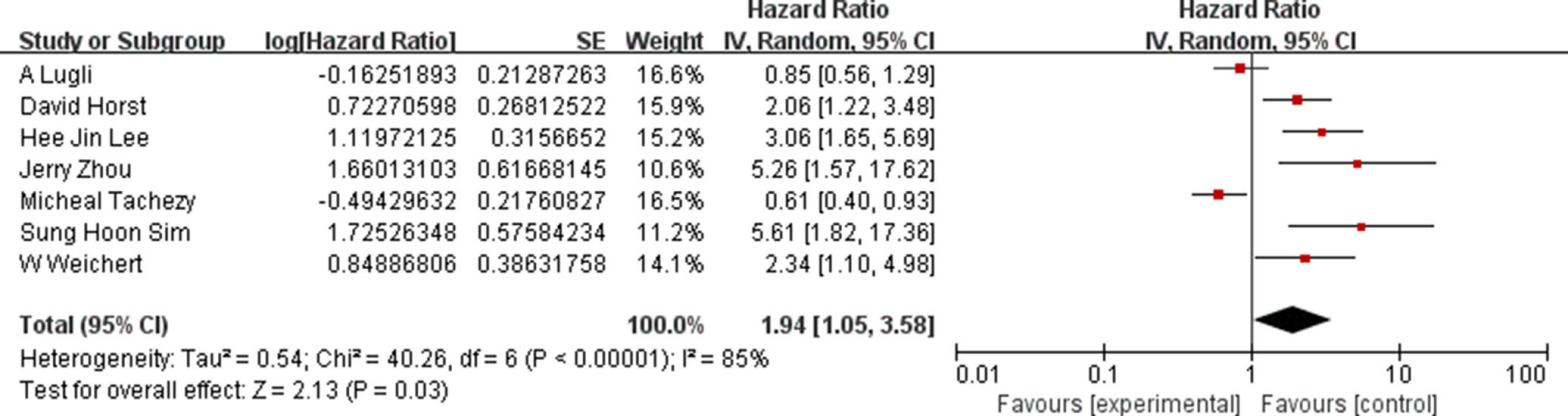
Association between high ALCAM expression and overall survival of CRC patients

**Figure 3:**
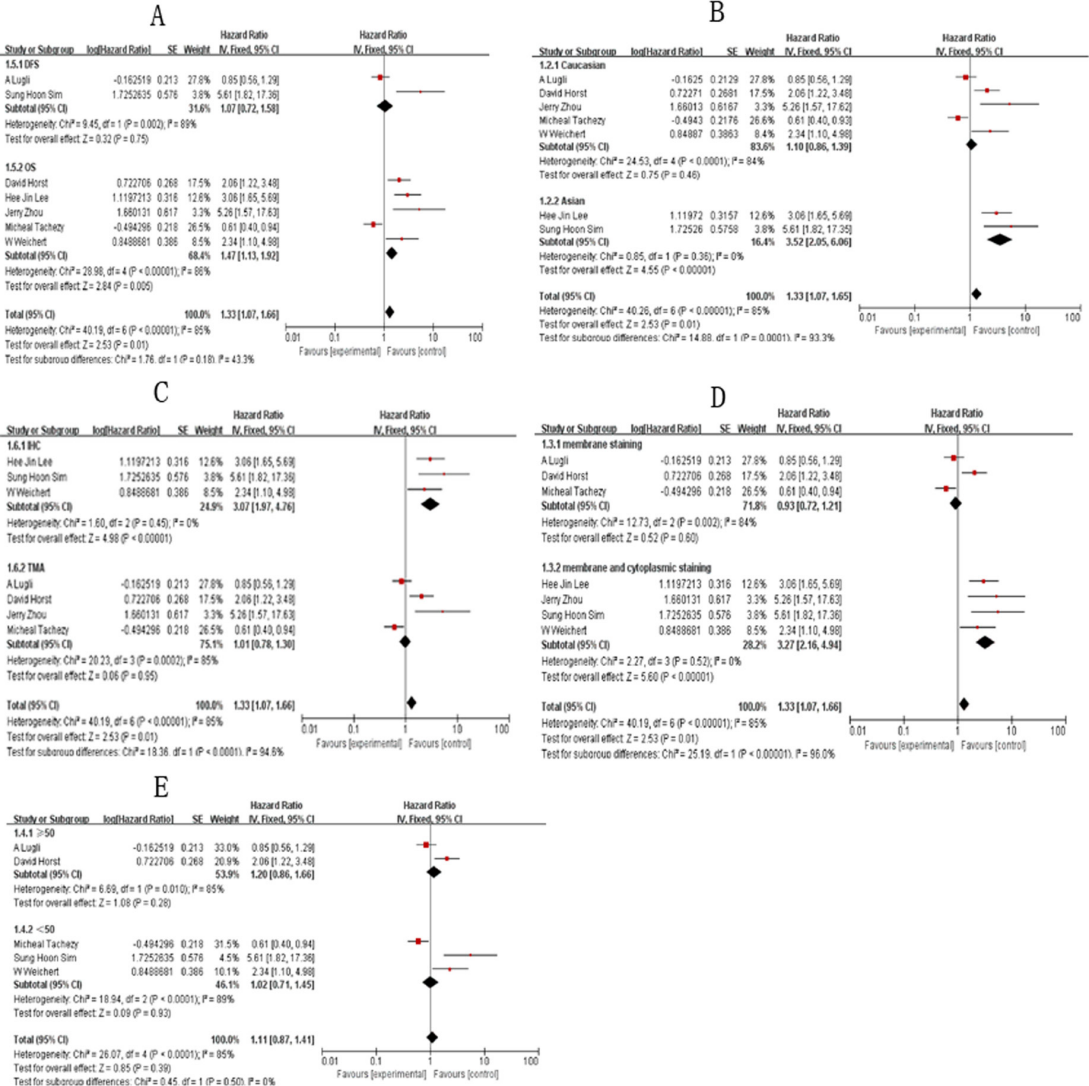
Subgroup analysis results showing association of ALCAM overexpression and overall survival of CRC patients (**A**) Results of subgroup analysis based on survival; (**B**) Results of subgroup analysis based on ethnicity; (**C**) Results of subgroup analysis based on testing methods; (**D**) Results of subgroup analysis based on staining pattern; (**E**) Results of subgroup analysis based on follow-up time.

The random-effects model was used to analyze the prognostic value of ALCAM expression in CRC based on heterogeneity test results (I^2^ = 85%, *P <* 0.00001). Higher ALCAM expression was associated with poor survival in CRC patients (HR = 1.94, 95% CI = 1.05–3.58, *P <* 0.00001; Figure 2). Figure 3 shows results of subgroup analysis based on five stratifications, namely, survival parameters, ethnicity, testing methods, staining pattern and follow-up time. Our analysis showed that high ALCAM expression was associated with poor overall survival of CRC patients (HR = 1.47, 95%CI = 1.12–1.92, *P <* 0.00001). However, no significant association was observed in disease-free survival (DFS) group. Further, our results showed that studies using IHC to detect ALCAM expression predicted poorer outcomes of CRC patients (HR = 3.07, 95%CI = 1.97–4.76, *P* = 0.0002) compared to studies using tissue microarray (TMA) to estimate ALCAM levels (HR = 1.01, 95%CI = 0.78–1.30, *P* = 0.45). Regarding ethnicity, high ALCAM expression predicted poor prognosis of Asian CRC patients (HR = 3.52, 95%CI = 2.05–6.06, *P <* 0.00001). Moreover, high ALCAM expression was associated with membrane and cytoplasmic staining (HR = 3.27, 95%CI = 2.16–4.94, *P* = 0.002). No significant association was observed between ALCAM expression and CRC patients based on follow-up time. The results of overall and subgroup analysis are summarized in Supplementary Table 1.

Among the 7 included studies, 5 focused on overall survival (OS) of patients and 2 on disease-free survival. Subgroup analysis on the 5 studies that focused on overall survival based on testing methods, ethnicity, staining pattern and follow-up time revealed that high expression of ALCAM predicted poor prognosis of CRC patients in membrane and cytoplasmic staining (HR = 3.0, 95%CI = 1.92–4.69, *P <* 0.00001), IHC method (HR = 2.75, 95%CI = 1.70–4.44, *P <* 0.00001), Asian ethnicity (HR = 3.06, 95%CI = 1.65–5.69, *P <* 0.00001) and ≥ 50 (HR = 2.06, 95%CI = 1.22–3.48, *P <* 0.00001) groups (Supplementary Figure 7; Supplementary Table 1). However, these results were limited by smaller sample sizes when the subjects were divided into subgroups. However, there was no heterogeneity among the 5 studies and therefore we predict that our results were accurate.

### Correlation of ALCAM to clinicopathological features

**Figure 4:**
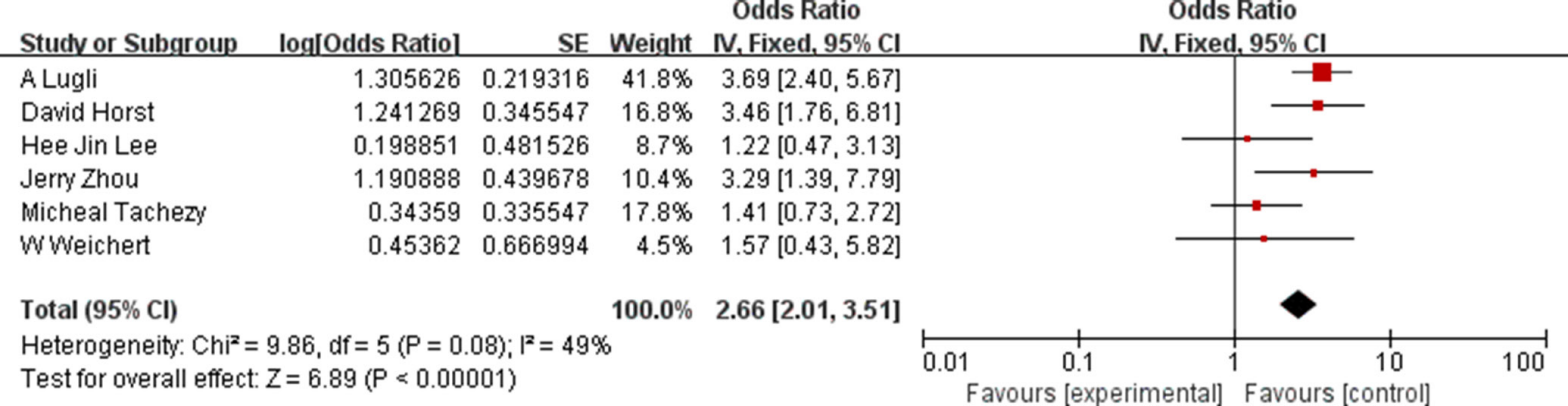
Association between ALCAM overexpression and tumor stage

Next, we analyzed the relationship between ALCAM expression and clinical features of CRC like tumor stage, nodal status, distant metastasis, tumor grade, age and gender. Six studies reported ALCAM expression in CRC patients with clinical tumor stages and our analysis showed that ALCAM overexpression was associated with advanced tumor stage [pooled OR (T3,T4 vs. T1,T2) = 2.66, 95%CI = 2.01–3.51, *P <* 0.0001; Figure 4). Moreover, higher ALCAM expression predicted positive nodal status in CRC patients (HR = 2.12, 95%CI = 1.61–2.82, *P <* 0.0001; Supplementary Figure 1). High expression of ALCAM was also significantly associated with distant metastasis (HR = 3.30, 95%CI = 2.21–4.91, *P <* 0.0001; Supplementary Figure 2). Analysis of 5 studies with 1826 cases that reported significance of ALCAM expression to tumor grade revealed that ALCAM overexpression was associated with the higher tumor grade (HR = 1.28, 95%CI = 1.00–1.62, *P* = 0.05; Supplementary Figure 3). Stratification analysis also showed association of high ALCAM expression with higher patient age and the poor risk of differentiation (HR = 1.29, 95%CI = 1.01–1.29, *P* = 0.05; Supplementary Figure 4). Although ALCAM expression was not associated with gender of patients, male CRC patients had lower ALCAM expression (HR = 0.94, 95%CI = 0.69–1.29, *P* = 0.72; Supplementary Figure 5). These results are summarized in Supplementary Table 2.

### Sensitivity analysis

**Figure 5:**
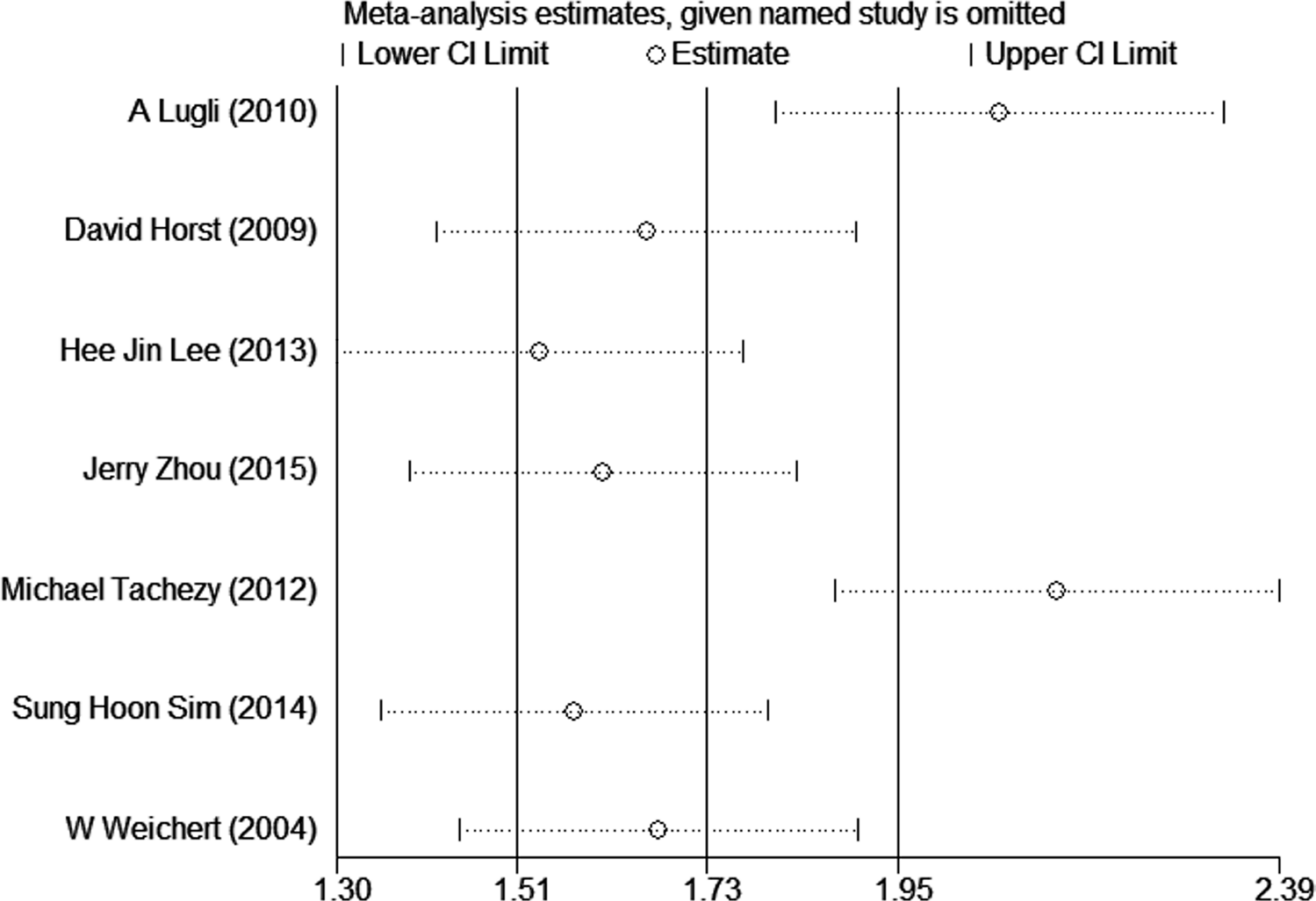
Sensitivity analyses among included studies

We conducted sensitivity analysis to assess the potential heterogeneity between studies by removing one study each time. As shown in Figure 5, no changes were observed in regard to the original meta-analysis suggesting that it was robust and consistent.

### Publication bias with trim and filling method

**Figure 6:**
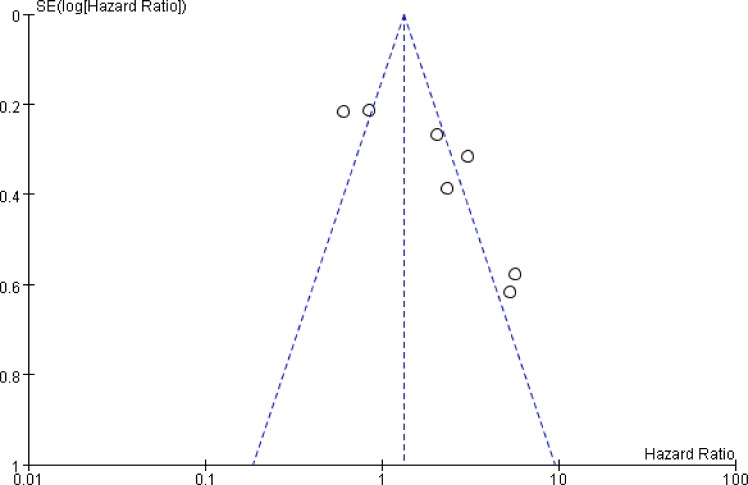
Funnel plot analysis of publication bias between high ALCAM expression and overall survival of CRC patients

**Figure 7:**
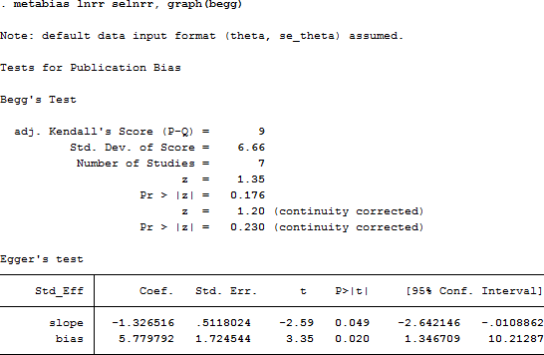
Begg's and Egger's tests of publication bias

We evaluated publication bias of the 7 studies using funnel plots (Figure 6), based on Begg's and Egger's test (Figure 7) and observed no obvious asymmetry (*P* = 0.230). Further, we conducted Trim and Filling analysis to robustly analyze publication bias and found that two more studies were necessary to eliminate publication bias thoroughly (variance = 0.578, *P* = 0.194, Supplementary Figure 6).

### Re-sampling statistics

Next, we used the bootstrap re-sampling procedure to generate 1000 re-sampling groups using overall ORs to estimate the accuracy and robustness of this meta-analysis. Our results demonstrated that the meta-analysis results were robust (pooled OR = 1.88, 95% CI = 1.87–1.88, *P* < 0.0001; Supplementary File 1).

## DISCUSSION

Numerous studies have investigated the association between ALCAM expression and the survival outcomes of CRC patients, but, the results have been contradictory. Tachezy et al. demonstrated that the high ALCAM expression was associated with the prolonged survival of CRC patients [21]. However, Weichert et al. reported that high ALCAM expression indicated poor overall survival in univariate analysis, contrary to previous findings [24]. Since these studies indicated the potential prognostic value of ALCAM in CRC despite the contradictory results, we conducted this meta-analysis to clarify the prognostic importance in CRC.

Our data indicated that overexpression of ALCAM predicted poor outcomes for clinical features like nodal status (positive vs. negative), tumor stage (T3, T4 vs. T1, T2), distant metastasis (M1 vs. M0), grade (grade3 vs. grade1,2) and age (> 60 vs. < 60). No correlation was found between ALCAM expression and gender in CRC patients. Also, high ALCAM expression was associated with poor survival outcomes of CRC patients using the random-effects model. Previously, Ni et al. demonstrated the prognostic value of CD166 (ALCAM) expression in digestive cancers [26]. However, only four studies on colorectal cancer (CRC) were enrolled to evaluate the association between ALCAM overexpression and clinical features. Further, the prognostic value of high ALCAM expression in CRC was not investigated [26]. Also, the clinicopathological characteristics and tumor-driving factors can vary for different types of cancers. Hence, we conducted this meta-analysis to further evaluate the prognostic value of ALCAM high expression in CRC patients. Compared to Ni's study, we systematically retrieved the studies from multiple databases with November 11, 2016 being the last search time. We also comprehensively analyzed the predictive value of ALCAM in evaluating the outcomes of CRC patients in subtypes.

Among the included studies, the staining patterns of ALCAM were highly heterogeneous. Three studies focused on membranous staining [21, 22, 25], whereas the other four studies considered both membrane and cytoplasmic staining. The four studies that analyzed both membrane and cytoplasmic staining indicated that high ALCAM expression reflected poor outcomes of CRC patients. In contrast, no significant association was found between ALCAM expression and clinical outcome by studies analyzing membrane staining only. Interestingly, in breast [27], ovarian [15] and oral [28] cancers, high membrane and cytoplasmic ALCAM expression is associated with poor outcomes.

Another source of heterogeneity in our analysis was the detecting methods of ALCAM expression. Three studies used immunohistochemistry and showed that high ALCAM expression indicated poor prognosis. These results were consistent with our findings. However, the 4 studies that used tissue microarray to analyze ALCAM expression showed no association. This could be because the antibody concentrations used for TMA ranged from 1:100 to 1:500 making it difficult to determine standard cut-off scores for the positive staining.

Although several molecular tumor markers were reported previously, none were amenable in clinical trials [29]. KRAS mutational analysis remains the only clinical biomarker for CRC [30]. Therefore, new biomarkers such as ALCAM need to be developed for clinical use. However, the mechanistic link between ALCAM and CRC is unknown. ALCAM may influence homotypic and heterotypic interactions between cells [31–33]. However, more mechanistic studies are needed. Besides, ALCAM can be activated by the KRAS mutations, thereby contributing to CRC tumorigenesis and metastasis [34]. Furthermore, activation of β-catenin by loss of adenomatous poluposis coil (APC) induces ALCAM expression.

There are some limitations in our meta-analysis. First, inconsistent cut-off scores among studies because of different detecting methods may contribute to heterogeneity. Second, the number of studies enrolled in our analysis is relatively small. Hence, our findings need to be confirmed by larger studies in future. Third, calculation errors could not be avoided when estimating data from Kaplan-Meier curves using Engage Digitizer 4.1. Finally, heterogeneity existed in some pooled outcomes and could not be resolved by subgroup analysis.

In conclusion, our meta-analysis shows that high expression of ALCAM is associated with poor outcome of CRC and predicts aggressive clinicopathological features. Therefore, ALCAM is a potential biomarker of clinical relevance for CRC patients.

## MATERIALS AND METHODS

### Literature research

We comprehensively searched the electronic databases PubMed, Cochrane Library, EMBASE, OVID and Web of Science using the following search keywords: ALCAM or CD166 and colorectal cancer or colorectal carcinoma or colon cancer or rectal cancer or CRC. Other bioinformatic sources such as Oncomine and TCGA (analyzed by cBioPortal) were also searched. The searches were conducted until November 11, 2016. The reference lists of all eligible studies were evaluated to avoid missing any potential data. Authors of articles were contacted if specific information was required.

### Selection criteria

The inclusion criteria were: 1) case-control and cohort studies that focused on the association between ACLAM expression and its prognostic significance in colorectal cancer; 2) studies with sufficient data to calculate the odds ratio (OR) or hazard ratio (HR) and the corresponding 95%CI; 3) the expression level of ALCAM was definitively tested by standard molecular assays; 4) adequate patients and control subjects were enrolled in studies (more than 40).

The exclusion criteria were: 1) letters, duplicate studies, reviews and case reports, 2) studies without sufficient data, 3) animal experiment studies or *in vitro* cell culture models. When multiple studies focused on the same population, the most recent one was used.

### Data extraction

Two authors (Y. Zhang and C. Qian) extracted the following data independently from the included studies: author names, publication years, ethnicity, country, sample size, mean age, distribution of age and gender, median follow-up time, survival condition, staining patterns, HR estimation and corresponding 95%CI, tumor node metastases, histological stage, differentiation degree and nodal status. We preferred multivariate analysis if both univariate and multivariate analysis were reported. If the studies provided only the Kaplan-Meier curves, data was extracted with the Engauge Digitizer 4.1 software. The data was thoroughly examined and any discrepancies were resolved by all authors.

### Quality assessment

The Newcastle-Ottawa Scale (NOS) was used to evaluate the quality of studies enrolled in our meta-analysis. The NOS system uses a star system based on three parts: selection (4 stars), comparability (2 stars) and outcome (3 stars). In this meta-analysis, studies that received 6 or more stars out of a maximum of 9 were regarded as high-quality. Any disagreements were resolved by all authors by discussion.

### Statistical analysis

The hazard ratios (HRs) and the corresponding 95% confidence intervals (CIs) were used to evaluate the prognostic value of ALCAM in colorectal cancer. Odds ratios (ORs) were used to assess the correlation between ALCAM expression and clinicopathological parameters (tumor node metastases, histological stage, differentiation degree and nodal status). Heterogeneity test was conducted to assess heterogeneity between studies using *Q*-test and I^2^ index. Random-effects or fixed-effects models were used based on the results of heterogeneity test. The statistical analysis was conducted by Review Manager Version 5.3 software (http://ims.cochrane.org/revman). Sensitivity analysis was performed by removing a study each time to assess the stability of our results. Begg's and Egger's asymmetry tests were performed by STATA software version 12.0 to identify potential publication biases in outcomes. Finally, the accuracy of the entities that were calculated in this meta-analysis was estimated by applying the bootstrap re-sampling procedure, which generated 1000 re-sampling groups to get robust and replicable results. Then overall ORs were analyzed for re-sampling statistics.

## SUPPLEMENTARY MATERIALS FIGURES AND TABLES




