# Supplementary material for: Meta-analysis indicating that high ALCAM expression predicts poor prognosis in colorectal cancer
Source: Oncotarget. 2017 May 8;8(29):48272–81. doi: 10.18632/oncotarget.17707 (PMC5564645; doi:10.18632/oncotarget.17707)
Supplement: Supplementary file 1 [file oncotarget-08-48272-s001.pdf]

Meta-analysis indicating that high ALCAM expression predicts poor prognosis in colorectal cancer

Supplementary Materials

Supplementary Table 1: Overall and subgroup meta-analysis of OS and ALCAM expression in colorectal cancer

Categories	Cohorts (*n*)	HR (95% CI)	I^2 (%)	*P* value	*Z*	*P* value
Overall	7 (2048)	1.94 (1.05–3.58)	85	< 0.00001	2.13	0.03
Survival conditions						
OS	5 (662)	1.47 (1.12–1.92)	86	< 0.00001	2.84	0.005
DFS	2 (1386)	1.07 (0.72–1.58)	89	0.002	0.32	0.75
Testing methods						
IHC	3 (319)	3.07 (1.97–4.76)	0	0.0002	4.98	< 0.00001
TMA	4 (1729)	1.01 (0.78–1.30)	85	0.45	0.06	0.95
Ethnicity						
Caucasian	5 (1840)	1.10 (0.86–1.39)	84	0.36	0.75	0.46
Asian	2 (208)	3.52 (2.05–6.06)	0	< 0.0001	4.55	< 0.00001
Staining pattern						
Membrane and cytoplasmic staining	4 (364)	3.27 (2.16–4.94)	0	0.002	5.60	< 0.00001
Membrane staining	3 (1684)	0.93 (0.72–1.21)	84	0.52	0.52	0.60
Follow-up time						
≥ 50 (month)	2 (1384)	1.20 (0.86–1.66)	85	0.010	1.08	0.28
< 50 (month)	3 (523)	1.02 (0.71–1.45)	89	< 0.00001	0.09	0.93

Supplementary Table 2: Summary of the meta-analysis data between ALCAM and clinicopathological characteristics

Subgroup	No. of studies	cases	OR	Heterogeneity		
			Pooled OR 95%CI	*P* Value	I^2 score (%)	*P* value
Tumor stage (T3,T4/T1,T2)	6	1936	2.66 (2.01–3.51)	< 0.0001	49	0.08
Nodal status (Positive/Negative)	6	1936	2.12 (1.61–2.82)	< 0.0001	0	0.43
Distant metastasis (M1/M0)	5	1826	3.30 (2.21–4.91)	< 0.0001	88	< 0.0001
Grade (grade3/grade1,2)	6	2003	1.28 (1.00–1.62)	0.05	77	< 0.0001
Age (> 60/< 60)	4	566	1.29 (1.01–1.66)	0.05	58	0.07
Gender(male/female)	4	551	0.94 (0.69–1.29)	0.72	6	0.36

Abbreviations: CI: confidence interval; OR: odds.

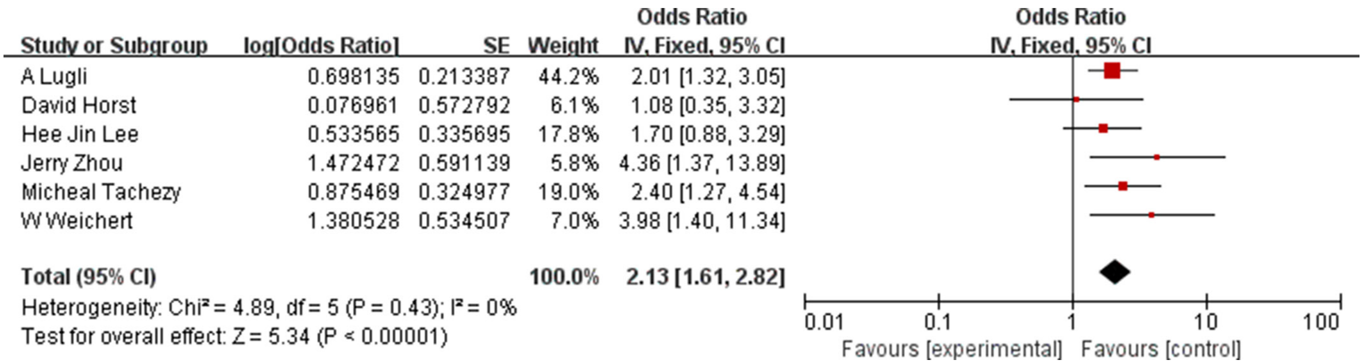

Supplementary Figure 1: Association between ALCAM overexpression and nodal status.

Study or Subgroup	log[Odds Ratio]	SE	Weight	Odds Ratio IV, Fixed, 95% CI
A Lugli	0.113329	0.30131	45.6%	1.12 [0.62, 2.02]
Hee Jin Lee	3.390473	0.521413	15.2%	29.68 [10.68, 82.47]
Jerry Zhou	1.706565	0.783314	6.7%	5.51 [1.19, 25.58]
Micheal Tachezy	1.175573	0.49908	16.6%	3.24 [1.22, 8.62]
W Weichert	1.98238	0.510349	15.9%	7.26 [2.67, 19.74]
**Total (95% CI)**			**100.0%**	**3.30 [2.21, 4.91]**

Heterogeneity: Chi² = 33.42, df = 4 (P < 0.00001); I² = 88%
Test for overall effect: Z = 5.86 (P < 0.00001)

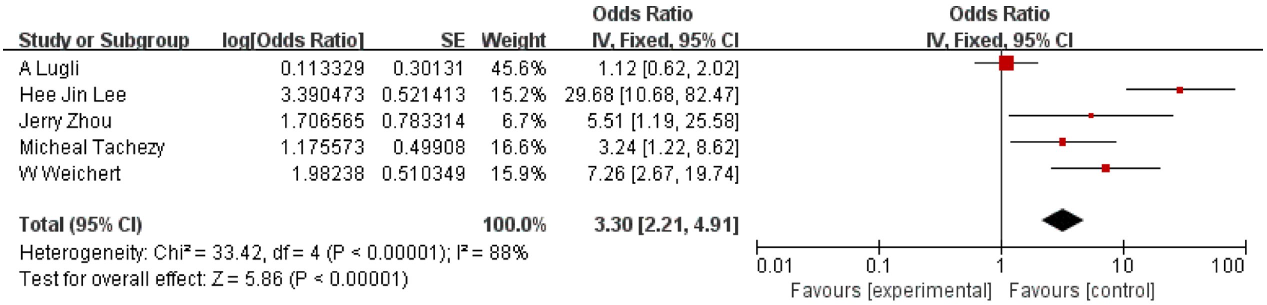

Supplementary Figure 2: Association between ALCAM overexpression and distant metastasis.

Study or Subgroup	log[Odds Ratio]	SE	Weight	Odds Ratio IV, Fixed, 95% CI
A Lugli	-0.30111	0.186636	43.3%	0.74 [0.51, 1.07]
David Horst	0.00995	0.615544	4.0%	1.01 [0.30, 3.37]
Hee Jin Lee	0.045929	0.33039	13.8%	1.05 [0.55, 2.00]
Micheal Tachezy	1.064711	0.326398	14.2%	2.90 [1.53, 5.50]
Sung Hoon Sim	0.854415	0.299627	16.8%	2.35 [1.31, 4.23]
W Weichert	0.934524	0.437637	7.9%	2.55 [1.08, 6.00]
**Total (95% CI)**			**100.0%**	**1.28 [1.00, 1.62]**

Heterogeneity: Chi² = 22.00, df = 5 (P = 0.0005); I² = 77%
Test for overall effect: Z = 1.99 (P = 0.05)

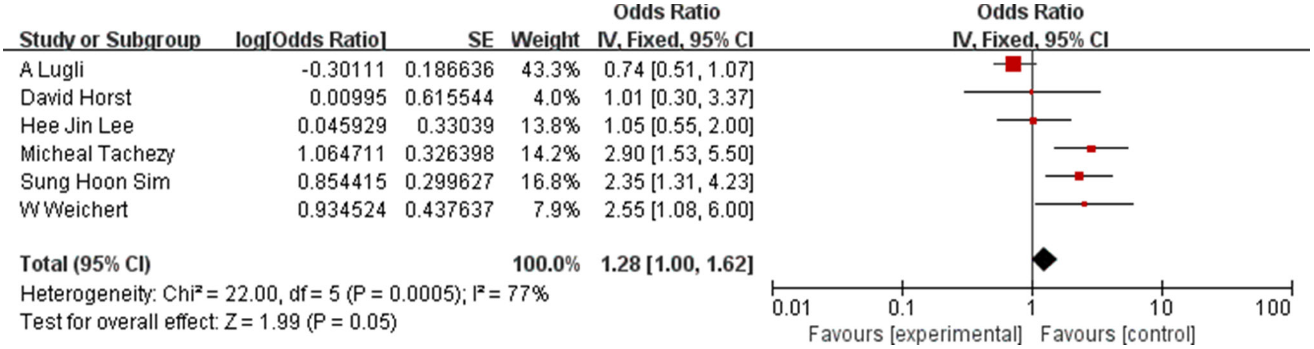

Supplementary Figure 3: Association between ALCAM overexpression and tumor grade.

Study or Subgroup	log[Hazard Ratio]	SE	Weight	Hazard Ratio IV, Fixed, 95% CI
David Horst	0.576613	0.354362	13.0%	1.78 [0.89, 3.56]
Jerry Zhou	-0.73397	0.565776	5.1%	0.48 [0.16, 1.45]
Micheal Tachezy	0.547543	0.209772	37.2%	1.73 [1.15, 2.61]
W Weichert	0.032467	0.191619	44.6%	1.03 [0.71, 1.50]
**Total (95% CI)**			**100.0%**	**1.29 [1.01, 1.66]**

Heterogeneity: Chi² = 7.17, df = 3 (P = 0.07); I² = 58%
Test for overall effect: Z = 2.00 (P = 0.05)

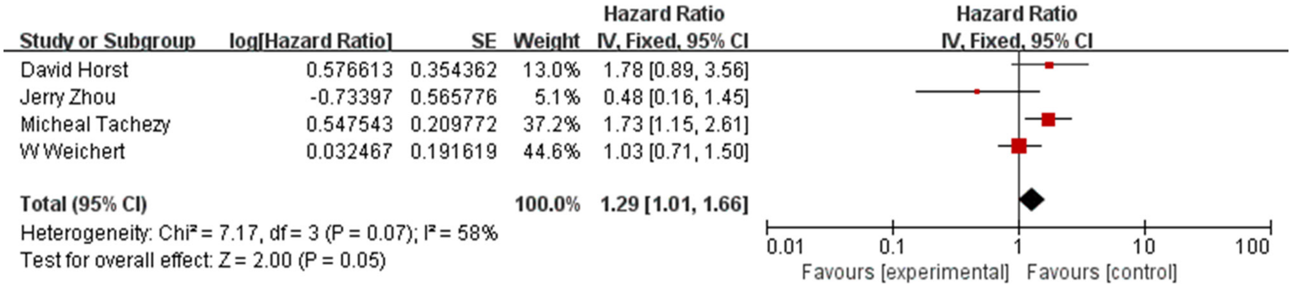

Supplementary Figure 4: Association between ALCAM overexpression and age.

Study or Subgroup	log[Odds Ratio]	SE	Weight	Odds Ratio IV, Fixed, 95% CI
David Horst	-0.13926	0.325843	24.6%	0.87 [0.46, 1.65]
Hee Jin Lee	0.193097	0.275279	34.5%	1.21 [0.71, 2.08]
Jerry Zhou	-0.96758	0.599841	7.3%	0.38 [0.12, 1.23]
Micheal Tachezy	-0.06188	0.278679	33.6%	0.94 [0.54, 1.62]
**Total (95% CI)**			**100.0%**	**0.94 [0.69, 1.29]**

Heterogeneity: Chi² = 3.19, df = 3 (P = 0.36); I² = 6%
Test for overall effect: Z = 0.36 (P = 0.72)

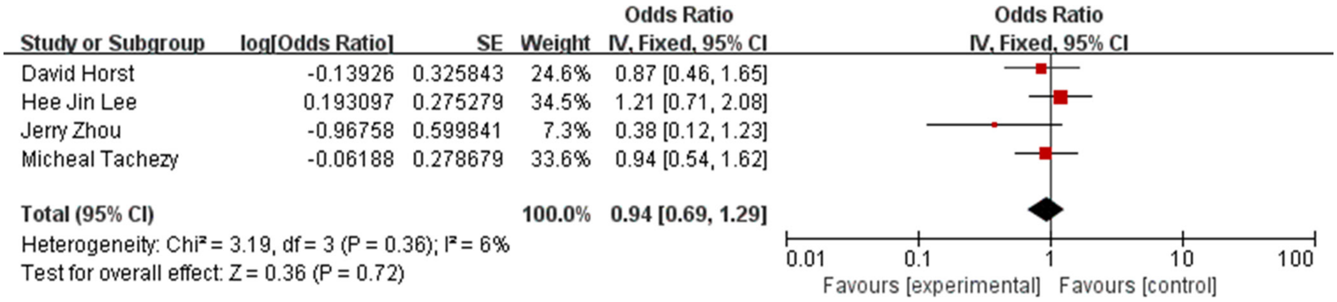

Supplementary Figure 5 : Association between ALCAM overexpression and gender.

Method	Pooled Est	95% CI Lower	95% CI Upper	Asymptotic z_value	Asymptotic p_value	No. of studies
Fixed	0.283	0.064	0.503	2.526	0.012	7
Random	0.663	0.052	1.275	2.126	0.033	

Test for heterogeneity: Q= 40.260 on 6 degrees of freedom (p= 0.000)
Moment-based estimate of between studies variance = 0.541

Trimming estimator: Linear
Meta-analysis type: Random-effects model

iteration	estimate	Tn	# to trim	diff
1	0.663	17	1	28
2	0.523	19	2	4
3	0.371	20	2	2
4	0.371	20	2	0

Filled
Meta-analysis

Method	Pooled Est	95% CI Lower	95% CI Upper	Asymptotic z_value	Asymptotic p_value	No. of studies
Fixed	0.201	-0.011	0.414	1.858	0.063	9
Random	0.376	-0.192	0.945	1.298	0.194	

Test for heterogeneity: Q= 48.317 on 8 degrees of freedom (p= 0.000)
Moment-based estimate of between studies variance = 0.578

Supplementary Figure 6: Analysis of publication bias by Trim and Filling method.

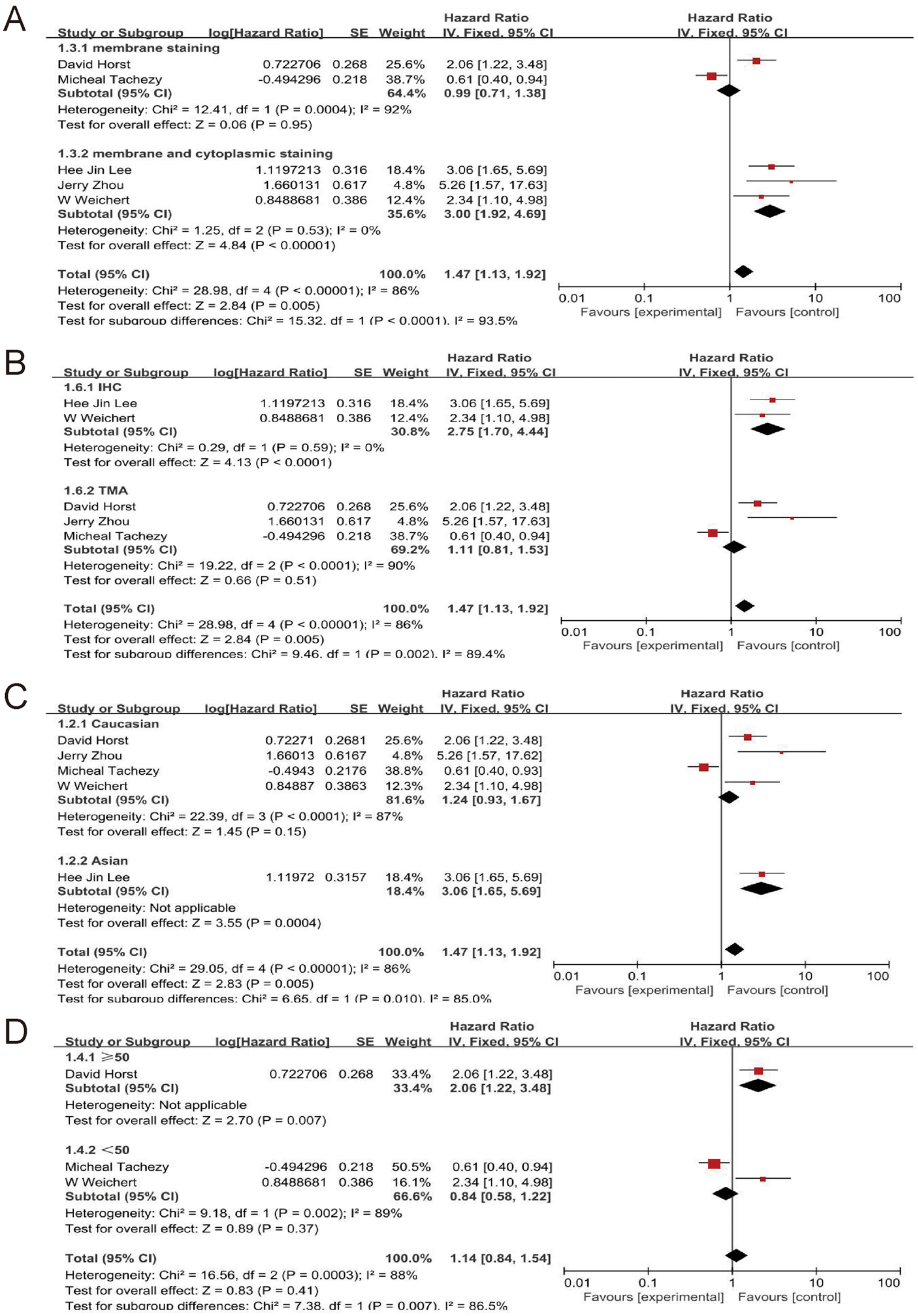

Supplementary Figure 7: Subgroup analysis regarding association between ALCAM overexpression and overall survival (OS) of CRC patients. (**A**) Results of subgroup analysis based on staining pattern; (**B**) Results of subgroup analysis based on testing methods; (**C**) Results of subgroup analysis based on ethnicity; (**D**) Results of subgroup analysis based on follow-up time.

Supplementary File 1: For sample type: 1 for high tumor stage and high expression of CD166, 2 for high tumor stage and low expression of CD166, 3 for low tumor stage and high expression of CD166, 4 for low tumor stage and low expression of CD166. See Supplementary _File_1
